# Hypnosis in the Perioperative Management of Breast Cancer Surgery: Clinical Benefits and Potential Implications

**DOI:** 10.1155/2016/2942416

**Published:** 2016-08-21

**Authors:** Arnaud Potié, Fabienne Roelants, Audrey Pospiech, Mona Momeni, Christine Watremez

**Affiliations:** Cliniques Universitaires Saint-Luc, Université Catholique de Louvain, Service d'Anesthésiologie, 10 Avenue Hippocrate, 1200 Brussels, Belgium

## Abstract

The aim of this review is to summarize data published on the use of perioperative hypnosis in patients undergoing breast cancer surgery (BCS). Indeed, the majority of BCS patients experience stress, anxiety, nausea, vomiting, and pain. Correct management of the perioperative period and surgical removal of the primary tumor are clearly essential but can affect patients on different levels and hence have a negative impact on oncological outcomes. This review examines the effect of clinical hypnosis performed during the perioperative period. Thanks to its specific properties and techniques allowing it to be used as complementary treatment preoperatively, hypnosis has an impact most notably on distress and postoperative pain. During surgery, hypnosis may be applied to limit immunosuppression, while, in the postoperative period, it can reduce pain, anxiety, and fatigue and improve wound healing. Moreover, hypnosis is inexpensive, an important consideration given current financial concerns in healthcare. Of course, large randomized prospective studies are now needed to confirm the observed advantages of hypnosis in the field of oncology.

## 1. Introduction

Breast cancer (BC) is an important public health concern. It is the most commonly encountered cancer among women worldwide and the leading cause of cancer-related mortality [1]. To improve outcomes, breast cancer surgery (BCS) is often required but can negatively affect patients on different levels, triggering psychological, physical, metabolic, neuroendocrine, inflammatory, and immunological changes [2–6]. During the perioperative period, anesthesiologists attempt to maintain homeostasis and induce a sense of comfort to offset the “stress reaction” frequently suffered by patients (including psychological and physiological stress) [4, 5, 7–9]. Relationships between the psychological and physiological aspects of cancer have been investigated by psychoneuroimmunology. Studies have shown that prolonged psychological stress probably impairs the immune response, which is one of the fundamental factors in the prognosis of oncology patients [10]. Thanks to the bidirectional communication between the neuroendocrine and immune systems, activation of one stimulates the other, which could constitute an additional strategy for perioperative clinical management of cancer patients [10].

Surgery is commonly required in the treatment of cancerous tumors, but this period is often considered critical for oncological outcomes because of the risk of tumor dissemination and possible depressive effects on immunity. Despite the growing array of available adjuvant therapies, tumor recurrence (local and distant dissemination) still remains the leading cause of death due to cancer [2, 3].

The aim of this review is to evaluate the contribution hypnosis can make during the perioperative period of BCS. A multimodal approach will be presented, as hypnosis can impact clinical, psychological, and pathophysiological aspects, which may all be involved in the outcome of cancer patients.

## 2. A Brief History of Hypnosis in Breast Cancer Management

Use of hypnosis in the treatment of BC started with a French surgeon, Cloquet, back in 1829 [11, 12]. This was the first documented application of hypnosis in BCS. Cloquet performed a mastectomy with axillary dissection, while the patient's physician, Dr. Chapelin, practiced something known as “magnetic sleep,” the word “hypnosis” not yet existing at that time [13].

After 1847, much major surgery, including breast surgery, was performed under hypnosis, but once ether and chloroform were made available to medical teams, these drugs became the standard of care for clinical anesthesia [14, 15]. Thus, hypnosis gradually disappeared from the “anesthetic environment.” Nevertheless, cases of BCS with hypnosis have been reported throughout the history of hypnosis [16].

During the 1950s, hypnosis was used as adjuvant therapy in the management of BC. In some cases, it was performed to reduce pain related to metastatic BC [17]. In the 1960s, Cangello showed the positive effect and power of posthypnotic suggestions to alleviate pain in cancer patients [18].

In 1983, Spiegel published the results of the first randomized controlled trial on the contribution of hypnosis to reducing pain in case of metastatic BC [19]. Since then, interest in hypnosis, initially focused on pain, has broadened to a general oncology treatment context.

Since 1992, hypnosis has been seen in operating rooms for procedures such as thyroidectomy, parathyroidectomy, and plastic surgery [20]. It can currently be used instead of general anesthesia for tumorectomies, quadrantectomies, and even mastectomies, in association with local or regional anesthesia [21, 22].

## 3. What Is Hypnosis? From Neurological Concepts to the Operating Room

It is difficult to provide a clear, uniform, and precise definition of hypnosis, as it depends upon the theoretical framework embraced by a given practitioner. However, the definition proposed by Montgomery characterizes hypnosis as “an agreement between a person designated as the hypnotist (e.g., healthcare professional) and a person designated as the client or the patient to participate in a psychotherapeutic technique based on the hypnotist providing suggestions for changes in sensation, perception, cognition, affect, mood, or behavior” [23]. This definition places emphasis on the relationship between hypnotist and patient, a necessary condition for anyone practicing hypnosis. More recently, the American Psychological Association (Division 30) proposed a definition of hypnosis as “a state of consciousness involving focused attention and reduced peripheral awareness characterized by an enhanced capacity for response to suggestion” [24].

From a clinical point of view, there are three basic phases during a hypnosis session, starting with induction, followed by therapeutic suggestions and concluded by emergence from the hypnotic state. During the induction phase, the therapist helps the patient to relax, imagine a peaceful scene, and become more focused on a “daydream.” During the second phase, the therapist provides the patient with suggestions. These suggestions are the key ingredients of hypnosis intended to treat specific symptoms or difficulties. For example, if the patient is prone to anxiety, the therapist may suggest that, during and/or following hypnosis, he/she will feel calmer or less bothered by those feelings. Emergence from hypnosis involves helping the patient recover a normal state of consciousness. The main and crucial element that distinguishes hypnosis from meditation or a relaxation session is use of suggestion [25].

When asked about their experience, patients describe alterations in body image, time distortion, dissociation, feelings of relaxation and peace, attentional focus, and increased positive affectivity, but diminished self-awareness and memory [26].

From a neurocognitive and neuroscientific perspective, hypnosis was thought to be mediated by the right cerebral hemisphere. This theory was supported by the discovery of hemispheric specialization in the 1970s, and the brain was artificially divided into the creative right and analytic left [27]. This simplistic standpoint has since been replaced by a more global approach that recognizes the complexity of the hypnotic experience, which involves the frontal lobes, prefrontal cortex, and anterior cingulate cortex of both hemispheres. Moreover, current interest in hypnosis by the neuroscientific community has two main angles. First, it contributes to a better understanding of the neurocognitive nature of hypnosis and hypnotic phenomena (e.g., by studying the neural activities accompanying hypnosis induction) [28]. Second, it allows use of hypnosis as a “research tool” to investigate normal psychological processes and neural substrates of a phenomenon (e.g., pain perception, color vision) [27–29].

From a neuroanatomical point of view, studies using voxel-based morphometry are able to assess hypnotic suggestibility-linked and hypnosis depth-related differences in local gray matter volume, especially in parts of the frontal, temporal, and occipital cortex [30].

During hypnosis, activation of regional cerebral blood flow distribution is different from activation triggered by evoking episodic memory [31] and by the resting state [32]. Different parts of the brain are involved, including occipital, parietal, precentral, premotor, and ventrolateral prefrontal areas, the anterior cingulate cortex, the thalamus, and the pontomesencephalic brainstem. Compared to “normal alertness” with mental imagery, a hypnotic state shows prominently decreased activity in the medial parietal cortex, particularly in the precuneus [31–33]. Precise data on neurophysiological findings are too specific to present here but have been well summarized by Vanhaudenhuyse et al. in a comprehensive review article [34].

While the neurobiological substrates of hypnosis have not been elucidated, results indicate that the attentional skills involved in hypnotizability may correlate with central dopaminergic activity [35].

Patients participate actively in the hypnotic experience through hypnotic techniques and suggestions; the hypnotic response is not effortless [36]. Hypnotic suggestions involve changes in the perception of elements and their cerebral interpretation, correlated with concomitant changes in neuroimages [37, 38].

During hypnosis, suggestions of analgesia lead to a higher pain threshold and/or decrease in pain perception. This effect has been demonstrated in a number of clinical trials and experimental pain studies on volunteers [34, 39]. This hypnoanalgesic effect appears to involve several pain pathways, such as the nociceptive reflexes, pain-related autonomic activity, and the supraspinal pain control system [34, 39, 40]. However, many factors can influence patient response to pain, including personality, cultural background, previous experiences, socioeconomic status, and psychosocial support.

Studies conducted by Faymonville et al. showed a significant reduction in pain scores, analgesic consumption, and postoperative nausea when patients underwent surgery with local anesthesia and hypnosis (hypnosedation) compared to general anesthesia [20, 40]. Similarly, in a prospective randomized study, Defechereux et al. found lower pain levels, less fatigue, improved recovery rates, and a decreased inflammatory response (IL-6) one day after surgery with use of hypnoanalgesia compared to general anesthesia [41]. Hypnosis techniques enhance intraoperative comfort and reduce anxiety, pain, and intraoperative requirements for anxiolytic and analgesic drugs, while ensuring optimal surgical conditions and faster recovery rates [22, 42]. In a prospective randomized trial, Lang et al. showed hypnosis to be beneficial during invasive medical procedures by decreasing pain and anxiety symptoms, improving hemodynamic stability, and shortening operating times [43].

To induce a hypnotic state during surgery, the therapist-patient relationship is crucial. Use of nonauthoritarian techniques promotes responsiveness to the therapist's suggestions, in addition to the patient's own responsiveness, driven by his/her desires and cooperation. Indeed, while mutual confidence is essential, the motivation and expectations of the patient are just as important as the specific means used to achieve the goals of this adjuvant therapy [40]. Moreover, at any time, the patient is free to change techniques (general anesthesia versus hypnosis), which intensifies the experience of self-mastery and self-control. It is the combination of all these elements that accounts for the increasing success of this psychological support during surgery. If confidence, motivation, and then cooperation are present, everybody can reach the hypnotic state.

## 4. Contribution of Hypnosis during the Three Phases of Surgery

### 4.1. Hypnosis during the Preoperative Period

Being diagnosed with cancer is a highly stressful experience for patients. BCS can be a particular source of anxiety and depression, as it impacts physical appearance, sexual identity, and life expectancy [44–46]. Other stress factors include specific fears; patients are often fearful of general anesthesia, anticipated pain, not waking up after surgery, and experiencing general postoperative discomfort, especially female patients [47].

In case of elective surgery, patients have time to prepare themselves. However, as the day of surgery approaches, they can experience higher anxiety and negative cognitions [48]. This causes emotional distress that may have a negative influence on postsurgery outcomes [44, 45, 48]. Individual distress variables, such as anxiety and catastrophizing thoughts, have been shown to be predictive of increased postoperative pain and chronic pain intensity [44, 49–51]. On the other hand, clinical studies reveal that preoperative psychological robustness (positive affectivity, dispositional optimism) predicts acute postoperative pain [52] and is an independent predictor of (and protector against) chronic postsurgical pain, even up to 4 months after surgery in a multivariate analysis [53].

The role of hypnosis in reducing distress related to medical procedures has been widely documented in the literature [54]. Schnur et al. showed a 15-minute presurgical hypnosis session to be an effective way of controlling preoperative distress in women awaiting diagnostic BCS, compared to a 15-minute presurgical attention control session [55]. Patients in the hypnosis group exhibited lower scores for preoperative emotional upset, depressed mood and anxiety, and better values on the visual analog scale of relaxation, than did attention controls. Montgomery's prospective randomized controlled trail illustrated the same perioperative benefits of a short presurgical hypnotic intervention [56]. Hypnosis was also found to mitigate self-reported postoperative pain (intensity and unpleasantness), discomfort, and emotional upset. Lang et al. showed that an adjuvant self-hypnosis relaxation session before the procedure decreased anxiety and pain levels during breast biopsy [57]. Moreover, preoperative hypnosis may yield positive postoperative effects, as demonstrated in a randomized study by Enqvist et al. [58]. They reported that preparation by self-hypnosis using recording tapes reduced postoperative nausea, vomiting, and analgesic requirements.

### 4.2. Hypnosis during Surgery

#### 4.2.1. Background and Immunity Considerations

Surgical removal of the primary tumor is the main treatment option for most cancers, but the perioperative period is a critical time for cancer evolution [3, 7, 59, 60]. Indeed, both surgical and medical factors play a role.

Surgical factors identified in human studies are related to tumor manipulation and tissue attrition, increasing shedding of malignant cells into the circulation, potentiating invasion of free malignant cells (mediated by matrix metalloproteinases and adhesion molecules), favoring a proangiogenic switch in micrometastases (VEGF), and increasing levels of growth factors (EGF) caused by tissue damage, and this encouraging local and distant recurrence [59, 60].

Medical factors are both endogenous and exogenous. Endogenous factors involve neuroendocrine (hypothalamic-pituitary-adrenal axis) and nervous system (nociceptive pathways and sympathetic system) loops [59]. Both perioperative psychological stress [60] and tissue injury [5] trigger numerous metabolic and hormonal changes, resulting in a “stress response” involving sympathetic, endocrine, and immunological effects. This response is characterized especially by increased secretion of catecholamine and pituitary hormones [5]. Stress hormones, specifically catecholamines, opioids, and glucocorticoids, were shown in animal models to causally promote metastatic progression (immunological and nonimmunological mechanisms) [60]. Moreover, during the perioperative period, natural killer (NK) cells, a component of the immune system, are particularly vulnerable, and the decline in their cytotoxic activity potentially exposes patients to increased oncological risk [59, 61]. In rats, stress caused by release of catecholamines was found to impair NK cell activity [62] and could therefore influence tumor progression [63].

Among exogenous factors, anesthesia and analgesic medications (e.g., opioids, nonsteroidal anti-inflammatory drugs (NSAIDs)) act directly and indirectly on immune cells [3, 59, 60, 64] and can potentially impact cancer recurrence or metastasis [3, 65, 66]. Morphine and its derivatives appear to depress NK cell activity in animal and human studies [59], while NSAIDs were shown to decrease the risk of BC in a retrospective human analysis [67]. Melamed et al. found that all anesthetics except propofol significantly reduced NK cell activity and increased lung tumor retention and lung metastasis in rats [64].

All these factors may well play a simultaneous role during the vulnerable period surrounding surgery, potentially affecting oncological outcomes. Control of these factors represents a window of opportunity to optimally manage cancer patients undergoing surgery. Maintaining their immune competence may therefore favor long-term remission [68].

#### 4.2.2. Do Hypnosis and Its Techniques Limit Immunosuppression?

*(a) Local and Locoregional Anesthesia*. Locoregional anesthesia during surgery also appears to have potential benefits in preventing cancer recurrence [69–71]. In experimental studies, a decline was observed in the spread of tumor cells and tumor recurrence in rats receiving regional anesthesia together with general anesthesia, compared to rats receiving general anesthesia alone [72]. Exadaktylos et al. retrospectively studied 129 patients who underwent a mastectomy with axillary dissection for BC. Recurrence-free and metastasis-free survival were significantly better in the group given a paravertebral block compared to those given only general anesthesia [73].

Furthermore, in experimental in vitro studies, local anesthetics appear to have specific cytotoxic effects on the tumor itself, which could negatively affect tumor progression [74–76]. Jose et al. [76] showed that levobupivacaine has specific cytotoxic effect on cancer cells viability inhibiting mitochondrial energy production.

*(b) Specific Effects of Hypnosis on Immunity*. Hypnosis practiced during surgery serves to comfort patients and reduce stress. It avoids the need for general anesthesia, with all its common side effects, and may therefore mitigate any possible immunosuppressive effects of general anesthesia.

Moreover, modest but reliable evidence suggests that psychological intervention can modulate the immune system [77]. During periods of stress, hypnosis can influence the dysregulation of immune cells (cellular and humoral immunity) [78–80]. This modification of the immune system is proportional to the level of decreased stress (CD3+ and CD4+ T lymphocytes) [81].

In the field of BC oncology, hypnosis as adjuvant treatment increases the number of NK cells and CD8+ T cells [82]. In a randomized controlled trial, Eremin et al. found significant differences in immunological modulation (activated T cells, lymphokine-activated killer cells, and mature T cells) in patients who underwent relaxation training and guided imagery techniques [83]. Indeed, higher numbers of cytotoxic T cells have been associated with longer survival durations in women with metastatic BC [84].

Spiegel et al. published two randomized prospective studies on the benefits of psychosocial therapy with hypnosis and survival of patients with metastatic BC. In the first study [85], patients in the treatment group (weekly supportive group therapy with self-hypnosis) lived significantly longer (about 18 months) than patients in the control group (routine oncological care). In the second study [86], they showed that overall survival did not differ between groups unless a specific factor was included, namely, the presence (+) or not (−) of the estrogen receptor (R) on the tumor. Patients who were R(−) had their survival expectations significantly improved by psychosocial treatment (educational materials and supportive group therapy training in learning self-hypnosis versus educational materials only), whereas no survival improvement was observed among R(+) patients.

*(c) Suggestions and Possible Implications*. During surgery, the anesthetist-hypnotherapist offers “suggestions,” with instantly favorable effects that may persist over time [87]. At the end of surgery, posthypnotic suggestions ensure continued comfort [43, 88], including positive emotions and antistress strategies, serving to enhance management in the postoperative period. These suggestions are very powerful and can act for prolonged periods of time. Such biobehavioral factors can significantly influence underlying cellular and molecular processes of malignant cells [89, 90] and may possibly impact oncologic outcomes.

#### 4.2.3. Concluding Remarks on the Perioperative Period

In summary, hypnosis eliminates the adverse effects of anesthetic drugs by avoiding their use, while potentiating the specific positive effects of hypnosis on immunity. Techniques that prevent stress limit the increase in catecholamine secretions and reduce the need for anesthetics and opioids and could therefore prove useful in decreasing the incidence of metastases. Hypnosis and its techniques can diminish the general stress response of the body, while having a positive impact on immunity, so, by combining local (or locoregional) anesthesia and hypnosis, we may limit perioperative immunosuppression and possibly influence the evolution of the disease. Prospective studies still need to be conducted, however, to confirm this speculation and demonstrate the real impact of perioperative hypnosis on immunity, and hence on survival, in BC patients.

### 4.3. Hypnosis during the Postoperative Period

The main adverse effects of postoperative surgical management of BC are nausea, vomiting, pain, and fatigue, and all of them can be mitigated by use of hypnosis and local anesthesia.

During the hypnotic state, “suggestions” involving analgesic strategies can help to reduce pain [88]. In turn, nociceptive and analgesic control may have an impact on cancer evolution. Poor management of pain in the postoperative period has been shown to have numerous consequences on potential long-term recurrence of metastases in rats [91].

Another long-term consequence of poor management of acute postoperative pain is the risk of it becoming chronic [92]. It has been shown that the only independent variable predicting severe pain two days after surgery is preoperative anxiety [50]. Patients with high levels of anxiety are at greater risk of acute postoperative pain and increased opioid consumption [44], and we know that 41% of patients exhibit signs of intense stress during the preoperative anesthesia evaluation. However, the intraoperative use of hypnosis can cut opioid consumption after surgery [22]. Practicing hypnosis means being vigilant to patient needs and communicating appropriately, especially during postoperative visits, where opioid consumption can be further reduced by using pain-free vocabulary [93].

Hypnosis can also have beneficial effects on postoperative wound healing. In a small randomized clinical trial, Ginandes et al. demonstrated the positive effects of hypnosis after mammoplasty [94]. Wound healing at both 1 and 7 weeks postsurgery was significantly improved in the hypnosis group.

A recent observational nonrandomized study compared groups of patients with the same characteristics but operated on either under hypnosis or general anesthesia. The number of postmastectomy lymphocele punctures was lower in the hypnosis group, as was the quantity of lymph fluid removed. Patients in the hypnosis group also showed a reduced incidence of asthenia induced by chemotherapy and radiotherapy and a lower rate of severe radiodermatitis. Finally, adherence to endocrine therapy was improved in the hypnosis group, while the incidences of hot flashes, joint or muscle pain, and asthenia were all significantly decreased [95]. Last but not least, using hypnosis for BCS has been shown to significantly shorten the hospital stay [22, 95].

## 5. Hypnosis and Cost Savings

Hypnosis is inexpensive, an important concern considering the current financial climate in hospitals and healthcare. Cost studies show a positive impact of using hypnosis in hospitals [20, 96–98] and for BC in particular [12, 56, 57, 99]. Block [99] reported the economic benefits of using hypnosis in patients undergoing breast biopsy. Their findings were then further extrapolated by Montgomery [12, 56]. It is important to note that surgery under hypnosis does not take longer than surgery under general anesthesia [41, 56, 57]. This is a key consideration given the high cost of occupancy of an operating room. As already stated, use of intraoperative hypnosis significantly shortens postoperative hospitalization [22, 95], yielding considerable saving. Moreover, when surgery is performed with a simple hypnoanalgesic technique, postanesthetic care is not even required. Hypnosis therefore reduces postanesthesia recovery times too [38].

## 6. Conclusions

Use of hypnosis in the context of BC is not new and many of its advantages have already been described in a meta-analysis on hypnosis in a surgical setting [100]. Suggestions made during hypnosis may affect patient outcomes improving various aspects like negative affectivity, emotional distress, pain, and analgesic medication consumption, as well as physiological indicators, such as nausea, vomiting, asthenia, lymphocele, and treatment, and recovery times. According to another review, the benefits of hypnosis are greater in the preoperative period [101]. Future research therefore investigate whether use of hypnosis during surgery can have any long-term benefits too. Anesthesiologists, in their role as perioperative physicians, should have knowledge of hypnosis and the power of suggestion to enhance the general clinical outlook during BCS. Indeed, it is important that anesthesiologists bear in mind the potential long-term impact of these techniques on the prognosis of patients.

Hypnosis can be used at every step of recovery. Moreover, during the perioperative period, it offers a unique patient-centered approach, creating a special bond between the patient and anesthesiologist. The latter affirms his/her role as a perioperative physician, endorsing the value of our work in the management of patients admitted for surgery.
